# Supplementary material for: Association between intraoperative end-tidal carbon dioxide and postoperative organ dysfunction in major abdominal surgery: A cohort study
Source: PLoS One. 2023 Mar 10;18(3):e0268362. doi: 10.1371/journal.pone.0268362 (PMC10004519; doi:10.1371/journal.pone.0268362)
Supplement: S1 File — (DOCX) [file pone.0268362.s002.docx]

**Supplemental data**

**Table S1.** Definitions of variables.

| Variables | Definitions |
| --- | --- |
| Age | In years, recorded in the anesthesia information management system |
| Male gender | recorded in the anesthesia information management system |
| ASAPS | 1 to 4, recorded in the anesthesia information management system |
| BMI | The body mass divided by the square of the body height, recorded in the anesthesia information management system |
| COPD | ICD-10 code of J43–44, recorded in the electronic medical record system |
| Albumin level of less than 30 g.l^-1^ | The most recent preoperative albumin level of less than 30 g.l^-1^, recorded in the electronic medical record system |
| Type of surgery | Grouped into six categories according to the operative site (colorectal, gastric, liver, pancreatic, esophageal, and complex) according to the procedure name recorded in the anesthesia information management system. “Complex” means a concomitant resection of two or more organs listed above |
| Laparoscopic surgery | Identified using the procedure name, recorded in the anesthesia information management system. Robotic surgery was treated as laparoscopic surgery |
| Emergency surgery | An emergency procedure, recorded in the anesthesia information management system |
| Epidural anesthesia | Defined as an epidural catheter placement documented in the anesthesia information management system, regardless of the duration of catheter placement, recorded in the anesthesia information management system |
| Duration of surgery | In hours, from skin incision to skin closure, recorded in the anesthesia information management system |
| MAP | The MAP was calculated by dividing the total intraoperative MAP by the duration from skin incision to skin closure for each patient. The intraoperative MAP, which was defined as the MAP from skin incision to skin closure, was extracted from the anesthesia information management system |
| Blood loss | In ml, recorded in the anesthesia information management system |
| Transfusion | In ml, recorded in the anesthesia information management system of the administration of red blood cells, fresh frozen plasma, and platelet during surgery |
| Infusion volume | In ml, recorded in the anesthesia information management system |
| Charlson Risk Index | Defined by the recent adoption of ICD-10 codes, recorded in the electronic medical record system |
| Mean RR, rpm | In rpm, defined as the total intraoperative RR divided by surgery duration, extracted from the anesthesia information management system |
| Mean FiO_2_, % | In %, defined as the total intraoperative FiO_2_ divided by surgery duration, extracted from the anesthesia information management system |
| Mean BIS | Defined as the total intraoperative BIS divided by surgery duration, extracted from the anesthesia information management system |
| Mean EtCO_2_ | Defined as the total intraoperative EtCO_2_ from skin incision to skin closure for each patient divided by surgery duration, extracted from the anesthesia information management system |
| Minimum EtCO_2_ | Defined as the minimum EtCO_2_ level of more than 20 mmHg from skin incision to skin closure, extracted from the anesthesia information management system |
| Maximum EtCO_2_ | Defined as the maximum EtCO_2_ level from skin incision to skin closure, extracted from the anesthesia information management system |

ASAPS, American Society of Anesthesiologists Physical Status; BMI, body mass index; COPD, chronic obstructive pulmonary disease; MAP, mean arterial pressure; ICD-10, International Classification of Diseases, Tenth Revision; RR, respiratory rate; FiO_2_, fraction of inspiratory oxygen; BIS, bispectral index; EtCO_2_, end-tidal carbon dioxide.

In this study, 83% of the patients who underwent major abdominal surgery used arterial catheters, recording the MAP every minute. Other patients used noninvasive blood pressure monitoring, recording MAPs every 2–5 min. If no arterial catheter was used, noninvasive blood pressure was substituted. We also used the MAP artifact removal algorithm, referenced in a previous study^28^: (1) out-of-range pressures: (a) systolic blood pressure (SBP) ≥ 300 or SBP ≤ 0 mmHg, (b) SBP ≤ diastolic blood pressure (DBP) + 5 mmHg or (c) DBP ≤ 0 mmHg or DBP ≥ 225 mmHg; (2) sudden change in SBP ≥ 80 mmHg within 1 min.

**Table S2.** Multivariate analysis of the relationship between EtCO_2_ and AKI.

|  | AKI (%) | Crude RR  (95% CI) | *P*-value | Adjusted  RR (95% CI)  Model 1‡ | *P*-value | Adjusted  RR (95% CI)  Model 2§ | *P*-value | P for  trend |
| --- | --- | --- | --- | --- | --- | --- | --- | --- |
| **Mean EtCO_2_** | | | | | |  |  | 0.536 |
| <35 mmHg | 98/1,195 (8.20%) | 1.08 (0.81–1.44) | 0.585 | 1.00 (0.76–1.33) | 0.947 | 0.98 (0.77–1.25) | 0.920 |  |
| 35–37 mmHg | 76/1,004 (7.57%) | 1 | – | 1 | – | 1 | – |  |
| 37–39 mmHg | 56/864 (6.48%) | 0.85 (0.61–1.19) | 0.361 | 0.96 (0.70–1.33) | 0.844 | 1.00 (0.76–1.31) | 0.987 |  |
| ≥39 mmHg | 60/1,108 (5.42%) | 0.71 (0.51–0.99) | 0.045 | 0.91 (0.66–1.26) | 0.591 | 0.83 (0.63–1.11) | 0.223 |  |
| **Minutes below EtCO_2_ 35 mmHg** | | | | | |  |  | 0.417 |
| Quartile value 1  (0–20 min) | 62/1,059 (5.85%) | 1 | – | 1 | – | 1 | – |  |
| Quartile value 2  (21–93 min) | 52/1,063 (4.89%) | 0.83 (0.58–1.19) | 0.326 | 0.77 (0.54–1.10) | 0.165 | 0.79 (0.57–1.10) | 0.172 |  |
| Quartile value 3  (94–223 min) | 74/1,038 (7.13%) | 1.21 (0.87–1.68) | 0.237 | 0.97 (0.70–1.33) | 0.868 | 1.04 (0.78–1.37) | 0.769 |  |
| Quartile value 4  (224–1,069 min) | 102/1,011 (10.09%) | 1.72 (1.27–2.33) | 0.000 | 1.01 (0.74–1.38) | 0.914 | 1.29 (0.99–1.70) | 0.059 |  |
| **Area under the threshold of EtCO_2_ 35 mmHg** | | | | | |  |  |  |
| Quartile value 1  (0–13 mm.Hg.min^-1^) | 60/1,048 (5.73%) | 1 | – | 1 | – | 1 | – | 0.504 |
| Quartile value 2  (14–102 mm.Hg.min^-1^) | 61/1,062 (5.74%) | 1.00 (0.70–1.41) | 0.985 | 0.97 (0.69–1.36) | 0.868 | 0.96(0.71–1.30) | 0.824 |  |
| Quartile value 3  (103–405 mm.Hg.min^-1^) | 74/1,044 (7.09%) | 1.23 (0.89–1.72) | 0.204 | 1.00 (0.73–1.38) | 0.972 | 1.01 (0.76–1.35) | 0.892 |  |
| Quartile value 4  (406–6,574 mm.Hg.min^-1^) | 95/1,017 (9.34%) | 1.63 (1.19–2.27) | 0.002 | 1.07 (0.78–1.47) | 0.638 | 1.19(0.90–1.58) | 0.198 |  |

Model 1‡: BMI, ASAPS 3 and above, laparoscopic surgery, type of surgery, epidural anesthesia, MAP, age, gender, emergency surgery, diabetes mellitus, congestive heart failure, ascites, hypertension, chronic kidney disease, surgery duration longer than 4 h, ischemic heart disease, cerebrovascular disease, albumin level of less than 30 g.l^-1^, and COPD.

Model 2§: age more than 56 years, gender, congestive heart failure, ascites, hypertension, chronic kidney disease, diabetes mellitus, and emergency surgery.

Abbreviations: AKI, acute kidney injury; EtCO_2_, end-tidal carbon dioxide; CI, confidence interval; RR, risk ratio; BMI, body mass index; ASAPS, American Society of Anesthesiologists Physical Status; MAP, mean arterial pressure; COPD, chronic obstructive pulmonary disease.

**Table S3.** Multivariate analysis of the relationship between EtCO_2_ and circulatory dysfunction.

|  | Circulatory dysfunction (%) | Crude  RR (95% CI) | P  value | Adjusted  RR (95% CI)  Model 1‡ | P  value | Adjusted  RR (95% CI)  Model 3§ | P  value | P for  trend |
| --- | --- | --- | --- | --- | --- | --- | --- | --- |
| **Mean EtCO_2_** | | | | | |  |  | 0.473 |
| <35 mmHg | 23/1,195 (1.92%) | 1.61 (0.80–3.22) | 0.178 | 1.61 (0.78–3.34) | 0.194 | 1.16 (0.79–3.28) | 0.183 |  |
| 35–37 mmHg | 12/1,004 (1.20%) | 1 | – | 1 | – | 1 | – |  |
| 37–39 mmHg | 10/864 (1.16%) | 0.96 (0.42–2.23) | 0.940 | 1.19 (0.53–2.69) | 0.660 | 1.20 (0.52–2.73) | 0.664 |  |
| ≥39 mmHg | 11/1,108 (0.99%) | 0.83 (0.36–1.87) | 0.655 | 1.24 (0.56–2.73) | 0.587 | 1.13 (0.51–2.53) | 0.751 |  |
| **Minutes below EtCO_2_ 35 mmHg** | | | | | |  |  | 0.327 |
| Quartile value 1  (0–20 min) | 8/1,059 (0.76%) | 1 | – | 1 | – | 1 | – |  |
| Quartile value 2  (21–93 min) | 9/1,063 (0.85%) | 1.12 (0.43–2.89) | 0.814 | 1.12 (0.46–2.71) | 0.796 | 1.02 (0.40–2.63) | 0.952 |  |
| Quartile value 3  (94–223 min) | 11/1,038 (1.06%) | 1.40 (0.56–3.47) | 0.464 | 0.90 (0.36–2.24) | 0.835 | 0.98 (0.39–2.42) | 0.966 |  |
| Quartile value 4  (224–1,069 min) | 28/1,011 (2.77%) | 3.66 (1.67–8.00) | 0.001 | 1.43 (0.63–3.26) | 0.385 | 1.47 (0.64–3.38) | 0.360 |  |
| **Area under the threshold of EtCO_2_ 35 mmHg** | | | | | |  |  | 0.094 |
| Quartile value 1  (0–13 mm.Hg.min^-1^) | 8/1,048 (0.76%) | 1 | – | 1 | – | 1 | – |  |
| Quartile value 2  (14–102 mm.Hg.min^-1^) | 7/1,062 (0.66%) | 0.86 (0.31–2.37) | 0.776 | 0.81 (0.31–2.09) | 0.664 | 0.80 (0.29–2.16) | 0.670 |  |
| Quartile value 3  (103–405 mm.Hg.min^-1^) | 12/1,044 (1.15%) | 1.50 (0.61–3.66) | 0.368 | 1.00 (0.42–2.34) | 0.995 | 1.00 (0.41–2.42) | 0.998 |  |
| Quartile value 4  (406–6,574 mm.Hg.min^-1^) | 29/1,017 (2.85%) | 3.37 (1.71–8.13) | 0.001 | 1.55 (0.69–3.47) | 0.286 | 1.66 (0.73–3.77) | 0.225 |  |

Model 1‡: BMI, ASAPS 3 and above, laparoscopic surgery, type of surgery, epidural anesthesia, MAP, age, gender, emergency surgery, diabetes mellitus, congestive heart failure, ascites, hypertension, chronic kidney disease, surgery duration longer than 4 h, ischemic heart disease, cerebrovascular disease, albumin level of less than 30 g.l^-1^, and COPD.

Model 3§: ischemic heart disease, congestive heart failure, cerebrovascular disease, diabetes mellitus, chronic kidney disease, surgery duration longer than 4 h, emergency surgery, and type of surgery.

Abbreviations: EtCO_2_, end-tidal carbon dioxide; CI, confidence interval; RR, risk ratio; BMI, body mass index; ASAPS, American Society of Anesthesiologists Physical Status; MAP, mean arterial pressure; COPD, chronic obstructive pulmonary disease.

**Table S4.** Multivariate analysis of the relationship between EtCO_2_ and respiratory dysfunction.

|  | Respiratory dysfunction (%) | Crude  RR (95% CI) | *P*-  value | Adjusted  RR (95% CI)  Model 1‡ | *P*-  value | Adjusted  RR (95% CI)  Model 4§ | *P*-value | P  For trend |
| --- | --- | --- | --- | --- | --- | --- | --- | --- |
| **Mean EtCO_2_** | | | | | |  |  | 0.270 |
| <35 mmHg | 16/1,195 (1.34%) | 2.68 (0.98–7.31) | 0.053 | 2.78 (1.05–7.32) | 0.038 | 2.41 (0.89–6.54) | 0.082 |  |
| 35–37 mmHg | 5/1,004 (0.50%) | 1 | – | 1 | – | 1 | – |  |
| 37–39 mmHg | 3/864 (0.35%) | 0.69 (0.16–2.90) | 0.621 | 1.04 (0.23–4.62) | 0.955 | 0.72 (0.17–3.02) | 0.661 |  |
| ≥39 mmHg | 8/1,108 (0.72%) | 1.44 (0.47–4.41) | 0.514 | 1.76 (0.57–5.44) | 0.320 | 1.49 (0.48–4.61) | 0.481 |  |
| **Minutes below EtCO_2_ 35 mmHg** | | | | | |  |  | 0.083 |
| Quartile value 1  (0–20 min) | 3/1,059 (0.28%) | 1 | – | 1 | – | 1 | – |  |
| Quartile value 2  (21–93 min) | 4/1,063 (0.38%) | 1.32 (0.29–5.92) | 0.710 | 1.25 (0.29–5.34) | 0.754 | 1.24 (0.27–5.56) | 0.772 |  |
| Quartile value 3  (94–223 min) | 8/1,038 (0.77%) | 2.72 (0.72–10.22) | 0.139 | 1.77 (0.47–6.71) | 0.396 | 2.30 (0.60–8.83) | 0.221 |  |
| Quartile value 4  (224–1,069 min) | 17/1,011 (1.68%) | 5.93 (1.74–20.19) | 0.004 | 2.65 (0.72–9.81) | 0.142 | 5.45 (1.62–18.27) | 0.006 |  |
| **Area under the threshold of EtCO_2_ 35 mmHg** | | | | | |  |  | 0.070 |
| Quartile value 1  (0–13 mm.Hg.min^-1^) | 2/1,048 (0.19%) | 1 | – | 1 | – | 1 | – |  |
| Quartile value 2  (14–102 mm.Hg.min^-1^) | 3/1,062 (0.28%) | 1.48 (0.24–8.84) | 0.667 | 1.38 (0.22–8.35) | 0.725 | 1.45 (0.25–8.43) | 0.675 |  |
| Quartile value 3  (103–405 mm.Hg.min^-1^) | 9/1,044 (0.86%) | 4.51 (0.97–20.86) | 0.053 | 3.10 (0.68–14.06) | 0.141 | 3.92 (0.83–18.38) | 0.083 |  |
| Quartile value 4  (406–6,574 mm.Hg.min^-1^) | 18/1,017 (1.77%) | 9.27 (2.15–39.87) | 0.003 | 3.76 (0.83–16.96) | 0.084 | 7.86 (1.91–32.36) | 0.004 |  |

Model 1‡: BMI, ASAPS 3 and above, laparoscopic surgery, type of surgery, epidural anesthesia, MAP, age, gender, emergency surgery, diabetes mellitus, congestive heart failure, ascites, hypertension, chronic kidney disease, surgery duration longer than 4 h, ischemic heart disease, cerebrovascular disease, albumin level of less than 30 g.l^-1^, and COPD.

Model 4§: age, emergency surgery, albumin level <30 g.l^-1^, blood urea nitrogen level ≥30 mg.dl^-1^, and COPD.

Abbreviations: EtCO_2_, end-tidal carbon dioxide; CI, confidence interval; RR, risk ratio; BMI, body mass index; ASAPS, American Society of Anesthesiologists Physical Status; MAP, mean arterial pressure; COPD, chronic obstructive pulmonary disease.

**Table S5.** Multivariable analysis of the relationship between EtCO_2_ and coagulation dysfunction.

|  | Coagulation dysfunction (%) | Crude  RR (95% CI) | *P*-value | Adjusted  RR (95% CI)  Model 1‡ | *P*-value | P  For trend |
| --- | --- | --- | --- | --- | --- | --- |
| **Mean EtCO_2_** | | | | | | 0.095 |
| <35 mmHg | 212/1,195 (17.74%) | 1.26 (1.03–1.53) | 0.019 | 1.09 (0.91–1.31) | 0.308 |  |
| 35–37 mmHg | 141/1,004 (14.04%) | 1 | – | 1 | – |  |
| 37–39 mmHg | 101/864 (11.69%) | 0.83 (0.65–1.05) | 0.132 | 1.00 (0.81–1.25) | 0.938 |  |
| ≥39 mmHg | 81/1,108 (7.31%) | 0.52 (0.40–0.67) | <0.001 | 0.89 (0.69–1.14) | 0.375 |  |
| **Minutes below EtCO_2_ 35 mmHg** | | | | | | 0.035 |
| Quartile value 1  (0–20 min) | 73/1,059 (6.89%) | 1 | – | 1 | – |  |
| Quartile value 2  (21–93 min) | 105/1,063 (9.88%) | 1.43 (1.07–1.90) | 0.014 | 1.27 (0.97–1.67) | 0.077 |  |
| Quartile value 3  (94–223 min) | 133/1,038 (12.81%) | 1.85 (1.41–2.44) | <0.001 | 1.21 (0.93–1.57) | 0.140 |  |
| Quartile value 4  (224–1,069 min) | 224/1,011 (22.16%) | 3.21 (2.50–4.12) | <0.001 | 1.37 (1.06–1.76) | 0.014 |  |
| **Area under the threshold of EtCO_2_ 35 mmHg** | | | | | | 0.018 |
| Quartile value 1  (0–13 mm.Hg.min^-1^) | 89/1,048 (8.49%) | 1 | – | 1 | – |  |
| Quartile value 2  (14–102 mm.Hg.min^-1^) | 88/1,062 (8.29%) | 0.97 (0.73–1.29) | 0.864 | 0.89 (0.68–1.17) | 0.429 |  |
| Quartile value 3  (103–405 mm.Hg.min^-1^) | 152/1,044 (14.56%) | 1.71 (1.33–2.19) | <0.001 | 1.15 (0.91–1.46) | 0.225 |  |
| Quartile value 4  (406–6,574 mm.Hg.min^-1^) | 206/1,017 (20.26%) | 2.38 (1.88–3.01) | <0.001 | 1.22 (0.96–1.54) | 0.091 |  |

Model 1‡: BMI, ASAPS 3 and above, laparoscopic surgery, type of surgery, epidural anesthesia, MAP, age, gender, emergency surgery, diabetes mellitus, congestive heart failure, ascites, hypertension, chronic kidney disease, surgery duration longer than 4 h, ischemic heart disease, cerebrovascular disease, albumin level of less than 30 g.l^-1^, and COPD.

Abbreviations: EtCO_2_, end-tidal carbon dioxide; CI, confidence interval; RR, risk ratio; BMI, body mass index; ASAPS, American Society of Anesthesiologists Physical Status; MAP, mean arterial pressure; COPD, chronic obstructive pulmonary disease.

**Table S6.** Multivariable analysis of the relationship between EtCO_2_ and liver dysfunction.

|  | Liver  dysfunction (%) | Crude  RR (95% CI) | *P*-value | Adjusted  RR (95% CI)  Model 1‡ | *P*-value | P  For trend |
| --- | --- | --- | --- | --- | --- | --- |
| **Mean EtCO_2_** | | | | | | 0.008 |
| <35 mmHg | 339/1,195 (28.37%) | 1.07 (0.93–1.23) | 0.302 | 1.04 (0.92–1.17) | 0.483 |  |
| 35–37 mmHg | 265/1,004 (26.39%) | 1 | – | 1 | – |  |
| 37–39 mmHg | 201/864 (23.26%) | 0.88 (0.75–1.03) | 0.120 | 0.94 (0.82–1.08) | 0.438 |  |
| ≥39 mmHg | 175/1,108 (15.79%) | 0.59 (0.50–0.70) | <0.001 | 0.86 (0.74–1.00) | 0.058 |  |
| **Minutes below EtCO_2_ 35 mmHg** | | | | | | 0.005 |
| Quartile value 1 (0–20 min) | 189/1,059 (17.85%) | 1 | – | 1 | – |  |
| Quartile value 2 (21–93 min) | 189/1,063 (17.78%) | 0.99 (0.82–1.19) | 0.968 | 1.08 (0.92–1.26) | 0.309 |  |
| Quartile value 3 (94–223 min) | 217/1,038 (20.91%) | 1.17 (0.98–1.39) | 0.077 | 1.00 (0.86–1.16) | 0.964 |  |
| Quartile value 4 (224–1,069 min) | 385/1,011 (38.08%) | 2.13 (1.83–2.48) | <0.001 | 1.20 (1.05–1.38) | 0.007 |  |
| **Area under the threshold of EtCO_2_ 35 mmHg** | | | | | | 0.011 |
| Quartile value 1 (0–13 mm.Hg.min^-1^) | 215/1,046 (20.52%) | 1 | – | 1 | – |  |
| Quartile value 2 (14–102 mm.Hg.min^-1^) | 186/1,062 (17.51%) | 0.85 (0.71–1.01) | 0.079 | 0.95 (0.82–1.11) | 0.562 |  |
| Quartile value 3 (103–405 mm.Hg.min^-1^) | 256/1,044 (24.52%) | 1.19 (1.01–1.40) | 0.029 | 1.09 (0.95–1.26) | 0.183 |  |
| Quartile value 4 (406–6,574 mm.Hg.min^-1^) | 323/1,017 (31.76%) | 1.54 (1.33–1.79) | <0.001 | 1.14 (1.00–1.31) | 0.043 |  |

Model 1‡: BMI, ASAPS 3 and above, laparoscopic surgery, type of surgery, epidural anesthesia, MAP, age, gender, emergency surgery, diabetes mellitus, congestive heart failure, ascites, hypertension, chronic kidney disease, surgery duration longer than 4 h, ischemic heart disease, cerebrovascular disease, albumin level of less than 30 g.l^-1^, and COPD.

Abbreviations: EtCO_2_, end-tidal carbon dioxide; CI, confidence interval; RR, risk ratio; BMI, body mass index; ASAPS, American Society of Anesthesiologists Physical Status; MAP, mean arterial pressure; COPD, chronic obstructive pulmonary disease

**Table S7.** Multivariable analysis of the relationship between EtCO_2_ and Organ dysfunction.

|  | Organ  Dysfunction(%) | Crude  RR (95% CI) | P  value | Adjusted  RR (95% CI) | P  value | Adjusted  RR (95% CI) | P  value |
| --- | --- | --- | --- | --- | --- | --- | --- |
| **Mean EtCO_2_** |  |  |  | **Model 1‡** |  | **Model 6‡** |  |
| Low EtCO_2_ | 498/1195(41.67%) | 1.33(1.22-1.45) | <0.001 | 1.11(1.03-1.20) | 0.006 | 1.08(1.00-1.17) | 0.041 |
| Normal EtCO_2_ | 930/2976(31.25%) | 1 | - | 1 | - | 1 | - |
| **Mean EtCO_2_** |  |  |  | **Model 1‡** |  | **Model 6‡** |  |
| Low EtCO_2_ | 473/1044(45.31%) | 1.29 (1.19-1.41) | <0.001 | 1.14(1.05-1.24) | 0.001 | 1.14 (1.05-1.23) | <0.001 |
| Normal EtCO_2_ | 848/2432(34.87%) | 1 | - | 1 | - | 1 | - |
| **Mean EtCO_2_** |  |  |  | **Model 1‡** |  | **Model 7‡** |  |
| Low EtCO_2_ | 335/711(47.12%) | 1.31(1.18-1.45) | <0.001 | 1.14(1.04-1.25) | 0.004 | 1.16(1.06-1.27) | 0.001 |
| Normal EtCO_2_ | 55/1548(35.85%) | 1 | - | 1 | - | 1 | - |

Model 1‡: BMI, ASAPS 3 and above, laparoscopic surgery, type of surgery, epidural anesthesia, and mean MAP, age, gender, emergency surgery, diabetes mellitus, congestive heart failure, ascites, hypertension, chronic kidney disease, surgery duration longer than 4 hours, ischemic heart disease, cerebrovascular disease, albumin level <30 g.l^-1^, and COPD

Model 5‡: BMI, ASAPS 3 and above, laparoscopic surgery, type of surgery, epidural anesthesia, and mean MAP, age, gender, emergency surgery, diabetes mellitus, congestive heart failure, ascites, hypertension, chronic kidney disease, surgery duration longer than 4 hours, ischemic heart disease, cerebrovascular disease, albumin level <30 g.l^-1^, COPD and PaCO_2_-EtCO_2_ gradient

Model 6‡: BMI, ASAPS 3 and above, laparoscopic surgery, type of surgery, epidural anesthesia, MAP, age, gender, emergency surgery, diabetes mellitus, congestive heart failure, ascites, hypertension, chronic kidney disease, surgery duration longer than 4 h, ischemic heart disease, cerebrovascular disease, albumin level of less than 30 g.l^-1^, COPD and intraoperative maximum lactate value(mmol.L^-1^)

Model 7‡: BMI, ASAPS 3 and above, laparoscopic surgery, type of surgery, epidural anesthesia, and mean MAP, age, gender, emergency surgery, diabetes mellitus, congestive heart failure, ascites, hypertension, chronic kidney disease, surgery duration longer than 4 hours, ischemic heart disease, cerebrovascular disease, albumin level <30 g.l^-1^, COPD and Median minute volume(ml.kg^-1^.min^-1^).

Abbreviations: EtCO_2_, end-tidal carbon dioxide; CI, confidence interval; RR, risk ratio; BMI, body mass index; ASAPS, American Society of Anesthesiologists Physical Status; MAP, mean arterial pressure; COPD, chronic obstructive pulmonary disease

**Table S8.** Respiratory parameters.

| Characteristics | All patients  (n = 2259) | Low EtCO_2_  (n = 711) | Normal EtCO_2_  (n = 1548) |
| --- | --- | --- | --- |
| Median plateau pressure, mmHg | 14 (11–16) | 13 (11–15) | 14 (12–16) |
| Median peak pressure, mmHg | 16 (14–18) | 15 (13–18) | 16 (14–18) |
| Median PEEP, mmHg | 2 (1–4) | 1 (1–3) | 2 (1–4) |
| Median tidal volume, ml.kg^-1^ | 8.50 (7.66–9.35) | 8.74 (7.91–9.57) | 8.37 (7.52–9.28) |
| Median minute volume, ml.kg^-1^.min^-1^ | 93 (83–104) | 93 (84–106) | 93 (82–104) |

Abbreviations: EtCO_2_, end-tidal carbon dioxide; PEEP, positive end expiratory pressure.

**Table S9. Subgroup analyses stratified by laparoscopic surgery**

|  | Organ Dysfunction(%) | Crude RR  (95% CI) | P  value | Adjusted  RR (95% CI) | P  value | P for  interaction |
| --- | --- | --- | --- | --- | --- | --- |
| Overall | 1438/4171(34.48%) | 1.33 (1.22–1.45) | <0.001 | 1.11 (1.03–1.20) | 0.006 |  |
| **Laparoscopic surgery** | | | | | | 0.003 |
| Yes | 541/2529(21.40%) | 1.48 (1.19–1.84) | <0.001 | 1.42(1.12–1.80) | 0.004 |  |
| No | 897/1642(54.60%) | 1.05 (0.86–1.28) | 0.608 | 1.14(0.91–1.42) | 0.237 |  |

Abbreviations: EtCO_2_, end-tidal carbon dioxide, CI, confidence interval; RR, risk ratio .
