# Supplementary material for: Association between intraoperative end-tidal carbon dioxide and postoperative organ dysfunction in major abdominal surgery: A cohort study
Source: PLoS One. 2023 Mar 10;18(3):e0268362. doi: 10.1371/journal.pone.0268362 (PMC10004519; doi:10.1371/journal.pone.0268362)
Supplement: Attachment — Submitted filename: Response to Reviewers_R2.docx [file pone.0268362.s003.docx]

Ref: PONE-D-22-12191R1

Academic Editor
PLOS ONE

Dear Prof. Jörn Karhausen:

We would like to thank all of you for providing us with valuable comments on our manuscript entitled “Association between Intraoperative End-Tidal Carbon Dioxide and Postoperative Organ Dysfunction in Major Abdominal Surgery: A Cohort Study” We have responded to all the reviewers' comments. Our itemized responses are listed below. We hope this revision process has remedied all the concerns related to the previous version of the manuscript. Changes or corrections of the original manuscript have been highlighted in red in the revised manuscript. The revised manuscript has also been checked and revised by a native English-speaking medical-editing professional.

We hope your response to our revised manuscript will be positive. Thank you once again for reviewing it.

Sincerely,

**Correspondence Author:**

Li Dong, M.D.Ph.D.

Department of Anaesthesia, Kyoto University Hospital, 54 Shogoin-Kawahara-cho, Kyoto 606-8507, JAPAN

Tel: +81-75-751-3433

E-mail: [dongli@kuhp.kyoto-u.ac.jp](mailto:dongli@kuhp.kyoto-u.ac.jp)

**Additional Editor Comments:**

**I thank the authors for the detailed responses and additional analyses following my comments. My only remaining point is the sentence (p.13) "In the subgroup analysis of laparoscopic versus open surgery, the point estimates of the risk ratio for organ dysfunction were exceeded than 1 but were particularly large in patients who underwent laparoscopic surgery (S9 Table)". The meaning of this sentence is not clear, please revise.**

**Response:** Thank you for this comment. We have rewritten the sentence(p.13) as

In the subgroup analysis of laparoscopic versus open surgery, the point estimates of the risk ratio for organ dysfunction were exceeded than 1 (S9 Table).

**Response to Additional Editor**

We wish to express our appreciation to the referee for the insightful comments that have helped us improve our manuscript significantly. We agree with all the comments and have incorporated them into the revised version of our manuscript (R2).

**Reviewers' comments:

Reviewer's Responses to Questions**

**Comments to the Author

1. If the authors have adequately addressed your comments raised in a previous round of review and you feel that this manuscript is now acceptable for publication, you may indicate that here to bypass the “Comments to the Author” section, enter your conflict of interest statement in the “Confidential to Editor” section, and submit your "Accept" recommendation.**

**Reviewer #1: (No Response)**

**2. Is the manuscript technically sound, and do the data support the conclusions?

The manuscript must describe a technically sound piece of scientific research with data that supports the conclusions. Experiments must have been conducted rigorously, with appropriate controls, replication, and sample sizes. The conclusions must be drawn appropriately based on the data presented.**

**Reviewer #1: No**

**3. Has the statistical analysis been performed appropriately and rigorously?**

**Reviewer #1: Yes**

**4. Have the authors made all data underlying the findings in their manuscript fully available?

The** [**PLOS Data policy**](http://www.plosone.org/static/policies.action#sharing) **requires authors to make all data underlying the findings described in their manuscript fully available without restriction, with rare exception (please refer to the Data Availability Statement in the manuscript PDF file). The data should be provided as part of the manuscript or its supporting information, or deposited to a public repository. For example, in addition to summary statistics, the data points behind means, medians and variance measures should be available. If there are restrictions on publicly sharing data—e.g. participant privacy or use of data from a third party—those must be specified.**

**Reviewer #1: No**

**5. Is the manuscript presented in an intelligible fashion and written in standard English?

PLOS ONE does not copyedit accepted manuscripts, so the language in submitted articles must be clear, correct, and unambiguous. Any typographical or grammatical errors should be corrected at revision, so please note any specific errors here.**

**Reviewer #1: No**

**6. Review Comments to the Author

Please use the space provided to explain your answers to the questions above. You may also include additional comments for the author, including concerns about dual publication, research ethics, or publication ethics. (Please upload your review as an attachment if it exceeds 20,000 characters)**

**Reviewer #1: Strobe Checklist is not appended and the paper is not structured accordingly.**

Response: Thank you for this comment. We have added the STROBE checklist. **Historical cohort study is not a scientific term to describe a study design. So as suggested, that study appears to be a consort study based on a medical records or registry. So the title " Historical cohort" is not specific.**

Response: Thank you for this comment. In compliance with STROBE [1], we refrained from using the term retrospective or historical and rewrote it as cohort study. We have rewritten the term " historical cohort study " as "cohort study". **Categorization of continuous variables introduces uncertainty in the definition of the primary endpoint variable. So this needs scientific justification, e.g. by refering to a regulatory guideline or consensus of medical discipline.**

Response: Thank you for this comment. The cutoff value for this study was determined to be 35 mmHg referring to consensus of medical discipline.:

(1) Clinically rational judgment: Hypocapnia, also known as hypocarbia, is a decrease in blood carbon dioxide (PaCO2) concentration below 35 mmHg, the normal reference range, as described in textbook.

(https://www.ncbi.nlm.nih.gov/books/NBK493167/#_NBK493167_pubdet_).

Furthermore, the normal value of end-tidal CO2 (EtCO2), the partial pressure of carbon dioxide at the end of expiration detected by capnometry, is usually 35-45 mmHg.

(https://www.ems1.com/ems-products/capnography/articles/5-things-to-know-about-capnography-Hr5ETRdXzCoU3fLH/)

(2) Cutoff according to a previous study: In a systematic review of parallel-arm randomized controlled trials(RCT) comparing hypercapnia and normocapnia in adult patients undergoing general anesthesia, 7 of 10 RCT definitions of cutoff were set at PaCO2 or EtCO2 as 35 mmHg, while other cutoffs were defined as EtCO2 33 mmHg,37 mmHg and PaCO2 36-40 mmHg[2].

Following your recommendation, we have revised the following information in the methods(Page6).

<Method

Dose effects were assessed using the mean EtCO_2_; patients were divided into two groups based on the cutoff level of 35 mmHg , widely used lower limit of normal PaCO_2_[2] [3].>

**7. PLOS authors have the option to publish the peer review history of their article (**[**what does this mean?**](https://journals.plos.org/plosone/s/editorial-and-peer-review-process#loc-peer-review-history)**). If published, this will include your full peer review and any attached files.


If you choose “no”, your identity will remain anonymous but your review may still be made public.

Do you want your identity to be public for this peer review? For information about this choice, including consent withdrawal, please see our** [**Privacy Policy**](https://www.plos.org/privacy-policy)**.**

**Reviewer #1: No**

1.	Vandenbroucke JP, von Elm E, Altman DG, Gøtzsche PC, Mulrow CD, Pocock SJ, et al. Strengthening the Reporting of Observational Studies in Epidemiology (STROBE): explanation and elaboration. Int J Surg. 2014;12(12):1500-24. Epub 2014/07/22. doi: 10.1016/j.ijsu.2014.07.014. PubMed PMID: 25046751.

2.	Petran J, Ansems K, Rossaint R, Marx G, Kalvelage C, Kopp R, et al. Effects of hypercapnia versus normocapnia during general anesthesia on outcomes: a systematic review and meta-analysis. Brazilian Journal of Anesthesiology (English Edition). 2022;72(3):398-406. doi: <https://doi.org/10.1016/j.bjane.2020.11.010>.

3.	Way M, Hill GE. Intraoperative end-tidal carbon dioxide concentrations: what is the target? Anesthesiol Res Pract. 2011;2011:271539. Epub 2011/11/24. doi: 10.1155/2011/271539. PubMed PMID: 22110496; PubMed Central PMCID: PMCPMC3202118.
