# Supplementary material for: Association between intraoperative end-tidal carbon dioxide and postoperative organ dysfunction in major abdominal surgery: A cohort study
Source: PLoS One. 2023 Mar 10;18(3):e0268362. doi: 10.1371/journal.pone.0268362 (PMC10004519; doi:10.1371/journal.pone.0268362)
Supplement: Attachment — Submitted filename: Response to Reviewers_R3.docx [file pone.0268362.s004.docx]

Ref: PONE-D-22-12191R1

Academic Editor
PLOS ONE

Dear Prof. Jörn Karhausen:

We would like to thank all of you for providing us with valuable comments on our manuscript entitled “Association between Intraoperative End-Tidal Carbon Dioxide and Postoperative Organ Dysfunction in Major Abdominal Surgery: A Cohort Study” We have responded to all the reviewers' comments. Our itemized responses are listed below. We hope this revision process has remedied all the concerns related to the previous version of the manuscript. There are no specific changes or revisions to the original manuscript as a result of this revision process.

We hope your response to our revised manuscript will be positive. Thank you once again for reviewing it.

Sincerely,

**Correspondence Author:**

Li Dong, M.D.Ph.D.

Department of Anaesthesia, Kyoto University Hospital, 54 Shogoin-Kawahara-cho, Kyoto 606-8507, JAPAN

Tel: +81-75-751-3433

E-mail: [dongli@kuhp.kyoto-u.ac.jp](mailto:dongli@kuhp.kyoto-u.ac.jp)

**Additional Editor Comments:**

**"In the subgroup analysis of laparoscopic versus open surgery, the point estimates of the risk ratio for organ dysfunction were exceeded than 1 (S9 Table)." Sorry to insist, but the sentence is still not clear. Do you mean ...the risk ratio exceeded 1?
Otherwise, all comments appear to be fully addressed. Thank you!**

Response: Thank you very much for your valuable comments.

In a subgroup analysis of laparoscopic versus open surgery, the adjusted risk ratio for laparoscopic surgery was 1.42 (adjusted risk ratio, 1.42; 95% confidence interval [CI], 1.12-1.80; p = 0.004), and for open surgery adjusted risk ratio, 1.14; 95% confidence interval [CI], 0.91-1.42; p = 0.237). Although the small sample size in the subgroup analysis makes it difficult to find significant differences, the point estimate of the risk ratio for organ dysfunction was exceeded than 1.

S9Table . Subgroup analyses stratified by laparoscopic surgery

|  | Organ Dysfunction(%) | Crude RR  (95% CI) | P  value | Adjusted  RR (95% CI) | P  value | P for  interaction |
| --- | --- | --- | --- | --- | --- | --- |
| Overall | 1428/4171(34.48%) | 1.33 (1.22–1.45) | <0.001 | 1.11 (1.03–1.20) | 0.006 |  |
| Laparoscopic surgery | | | | | | 0.003 |
| Yes | 541/2529(21.40%) | 1.48 (1.19–1.84) | <0.001 | 1.42(1.12–1.80) | 0.004 |  |
| No | 897/1642(54.60%) | 1.05 (0.86–1.28) | 0.608 | 1.14(0.91–1.42) | 0.237 |  |
